# Prevalence and determinants of chronic kidney disease in northeast of Iran: Results of the Golestan cohort study

**DOI:** 10.1371/journal.pone.0176540

**Published:** 2017-05-03

**Authors:** Sadaf G. Sepanlou, Hamid Barahimi, Iraj Najafi, Farin Kamangar, Hossein Poustchi, Ramin Shakeri, Monir Sadat Hakemi, Akram Pourshams, Masoud Khoshnia, Abdolsamad Gharravi, Behrooz Broumand, Ali Nobakht-Haghighi, Kamyar Kalantar-Zadeh, Reza Malekzadeh

**Affiliations:** 1 Digestive Diseases Research Center, Digestive Diseases Research Institute, Tehran University of Medical Sciences, Tehran, Iran; 2 Non-Communicable Diseases Research Center, Shiraz University of Medical Sciences, Shiraz, Fars, Iran; 3 Department of Nephrology, Shariati Hospital, Tehran University of Medical Sciences, Tehran, Iran; 4 Department of Public Health Analysis, School of Community Health and Policy, Morgan State University, Baltimore, Maryland, United States of America; 5 Golestan Research Center of Gastroenterology and Hepatology, Golestan University of Medical Sciences, Gorgan, Iran; 6 Academy of Medical Sciences, Tehran, Iran; 7 Division of Nephrology & Hypertension, School of Medicine, University of California Irvine, Irvine, California, United States of America; 8 Veterans Affairs (VA) Long Beach Healthcare System‎, Long Beach, California, United States of America; 9 Department of Epidemiology, UCLA Fielding School of Public Health, Los Angeles, California, United States of America; Universita degli Studi di Perugia, ITALY

## Abstract

**Background:**

The burden of chronic kidney disease (CKD) is increasing globally in particular in fast emerging economies such as Iran. Population-based studies on prevalence of CKD in Iran are scarce. The objective of the current study was to explore the prevalence and determinants of CKD in the setting of Golestan Cohort Study (GCS), the largest prospective cohort in the Middle East.

**Methods:**

In this observational study, 11,409 participants enrolled in the second phase of GCS were included. Sex, age, literacy, residence, anthropometric measurements, smoking, opium use, self-reported history of cardiovascular diseases (heart disease and/or stroke), hypertension, diabetes, and lipid profile were the predictors of interest. The outcomes of interest were eGFR and CKD defined as eGFR< 60 ml/min/1.73m^2^.

**Results:**

Mean (SD) of GFR was 70.0 ± 14.7 ml/min/1.73m^2^ among all participants, 68.2 ± 14.2 among women, and 72.0 ± 15.0 among men. Prevalence of CKD was 23.7% (26.6% in women, 20.6% in men). The prevalence of CKD stages 3a, 3b, 4, and 5 were 20.0%, 3.3%, 0.4% and 0.1%, respectively. Female sex, older age, urban residence, history of CVD, hypertension or diabetes, larger body mass and surrogates of body fat and opium use were all associated with CKD. Opium had a significant positive association with CKD in adjusted model. All anthropometric measurements had positive linear association with CKD. Being literate had inverse association. Sex had significant interaction with anthropometric indices, with higher odds ratios among men compared with women. A significantly high association was observed between the rate of change in waist circumference and systolic blood pressure with risk of CKD.

**Conclusion:**

One in four people in this cohort had low eGFR. Obesity and overweight, diabetes, hypertension, and dyslipidemia are major risk factors for CKD. Halting the increase in waist circumference and blood pressure may be as important as reducing the current levels.

## Introduction

The burden of Chronic Kidney Disease (CKD) has been increasing globally [1], primarily in developing countries and emerging economies [2], while in developed nations the trend is either stable or somewhat decreasing. The Global Burden of Disease (GBD) study shows that mortality due to CKD in Iran increased from less than 1% in 1990 to over 2% in 2013, and low glomerular filtration rate (GFR) is among main risk factors of mortality and morbidity in Iran. [3–5]

The rising prevalence of CKD in Iran calls for urgent action. As a first step, the burden of CKD and its trend should be quantified. Due to scarcity of population-based studies and lack of resources, there are currently very few reports on prevalence of CKD in Iran. [6–11]. Early detection of CKD can significantly help prevent its progress and thus avoid huge costs of end stage renal disease that will be imposed on the society in future. As CKD is asymptomatic, its detection in its early stages is difficult if not impossible.

Apart from the scarcity of population based studies on prevalence of CKD in Iran, the determinants of CKD are also very rarely studied in our country. Substantial differences exist between the Iranian culture and the culture and life style in developed countries. While most of the good evidence on CKD and its prevalence and determinants comes from data-rich developed countries, evidence in developing countries such as Iran is quite scarce. Evidence-based policy making is a necessity in Iran and it is of outmost importance to develop policies that are tailored to specific needs, culture, and life style of Iranians.

In short, high quality population based studies on CKD are a necessity in Iran. In this study, we used data from the Golestan Cohort Study (GCS), the largest cohort in Iran and the entire Middle East region [12], to study the prevalence and determinants of CKD in an Iranian community. This is in fact the main merit of the current study, which is among the first in Eastern Mediterranean region.

## Materials and methods

### Population and study design

The details of the GCS have been described in previous studies.[12–14] In the baseline phase, all residents between 40 and 75 years old in 326 villages in Golestan Province and a sample of residents in Gonbad city were recruited from 2004 to 2008. The participants in Gonbad city were recruited through cluster random sampling that was proportional to size. The only exclusion criteria were age out of the range of 40 to 75 years, unwillingness to participate, and being a temporary resident in Golestan province. Thus, the cohort is a population-based sample of residents in Golestan province in North East of Iran. Briefly, 50,045 participants were recruited and annually actively followed up for occurrence of death or any major disease. At baseline, demographic data and existing major diseases, lifestyle risk factors (smoking, and opium use), medication history, as well as blood pressure and anthropometric measurements were recorded but serum biomarkers were not measured.

A total of 11,409 participants randomly selected from the whole study population underwent repeated measurement from 2010 to 2012. During the repeated measurement step, in addition to all of the above mentioned data, serum biomarkers were also measured. As serum creatinine was measured only in repeated measurement, only data from the 11,409 participants who underwent repeated measurements were used for the current analysis of GFR to determine the prevalence of CKD.

The potential determinants of interest included sex, age, literacy (literate vs. illiterate), residence (urban vs. rural), socio-economic status, anthropometric measurements, tobacco smoking, opium use, self-reported history of cardiovascular diseases (heart disease and/or stroke), hypertension, diabetes, and lipid profile at repeated measurement.

Data on past or current smoking were recorded. Data on substance use have been collected in detail in GCS. Opium is the major type of substance used in Iran and in Golestan province. In this study, past or current history of regular opium use, either by ingestion or inhalation, has been included in analyses.[14]

This study was approved for ethical considerations by the institutional board of Digestive Diseases Research Institute affiliated to Tehran University of Medical Sciences. Written informed consent was obtained from all participants in GCS, both in the baseline main phase and in repeated measurement phase. We obtained the written consent after we fully explained the process and aims of the study to all participants. Additionally, the consent to participate in the study was obtained from illiterate participants after they visited the study center and the procedures of the study were explained to them in detail.

### Definition of exposures

Physical exam including anthropometric and blood pressure measurements were performed by trained health personnel. Height, weight, waist and hip circumference were measured with light clothing. Blood pressure was recorded after 5 minutes of rest and in sitting position, twice from each arm with 10-minute intervals, using Richter auscultatory sphygmomanometers. The calculated average systolic and diastolic blood pressure were taken as mean systolic and diastolic blood pressures respectively. Hypertension was defined as having any of the following risks: systolic blood pressure (SBP) > = 140 mmHg, diastolic blood pressure (DBP) > = 90 mmHg, self-reporting of hypertension, or intake of anti-hypertensive medications.

Diabetes was defined as self-reported diabetes or intake of blood glucose lowering medications or having an FBS> = 126 mg/dL.

High Density Lipoprotein (HDL) was included in analyses as being high or low. Low HDL was defined as less than 40 mg/dL in men and less than 50 mg/dL in women.

Additional exposures of interest included levels of SBP, DBP, and anthropometric measurements at baseline in addition to rate of their change from baseline until repeated measurement.

### Kidney disease related variables

Outcomes of interest included GFR and CKD at repeated measurement. Serum creatinine levels were measured according to the standard colorimetric Jaffe-Kinetic reaction method (Pars Azmon Inc., Iran), with an inter assay CV of 2.5%, an intra-assay CV of 1.9%, and a sensitivity of 0.2 mg/dL. The assay range was 18–1330 mol (0.2–15 mg/dL). Assay performance was checked after every 30 tests using the control serum, TrueLab N (Lot. no. 11382; Pars Azmon, Inc., Iran) for normal ranges and TrueLab P (Lot. no. 11383; Pars Azmon, Inc., Iran) for pathological ranges. The assay was not traceable to isotope dilution mass spectroscopy (IDMS).

Estimated glomerular filtration rate (eGFR) was calculated by the traditional 4-variable Modification of Diet in Renal Disease (MDRD) equation [15–17]:

$$\text{GFR}\;\left[\text{mL}/\text{min}/1.73\;\text{m}2\right]\;=\;186\;\times \;{\left(\text{serum}\;\text{creatinine}\;\left[\text{mg}/\text{dL}\right]\right)}^{-1.154}\;\times \;{\left(\text{age}\;\left[\text{years}\right]\right)}^{-0.203}\;\times \;0.742\;\left(\text{if}\;\text{female}\right)\;\times \;1.212\;\left(\text{if}\;\text{African-American}\right).$$

This equation is valid for use with creatinine assays that are not IDMS traceable. We defined CKD in this study by one measurement of creatinine serum and eGFR less than 60 ml/min/1.73m^2^. Additionally, eGFR was divided into 6 stages: eGFR > = 90 in stage 1, > = 60 and <90 in stage 2, > = 45 and <60 in stage 3a, > = 30 and <45 in stage 3b, > = 15 and <30 in stage 4, and <15 in stage 5.

### Statistical methods

In the first step, our analyses were all done in the repeated measurement phase of the study. The covariates belong to this phase and the serum creatinine has also been measured only once in this phase. The current study is a cross-section of the repeated measurement (2010 to 2012). So we have in fact estimated the prevalence of low eGFR (< 60 ml/min/1.73m2) and its determinants in one cross-section.

In the second step of the analyses, we used SBP, BMI, and waist circumference and other covariates from the baseline and also calculated the rate of change in SBP, BMI, and waist circumference from the baseline till the repeated measurement. The outcome is again the serum creatinine in the repeated measurement.

In both steps of the analysis, univariate and multivariate logistic regression models were used for CKD as a dichotomous response variable. P-values less than 0.05 were considered statistically significant. Restricted cubic splines were used to explore the association of various anthropometric measurements with CKD. All statistical analyses were done using Stata statistical software version 13 (StataCorp, College Station, TX).

## Results

During the repeated measurement phase of GCS, 11,409 participants (5,996 women and 5,413 men) were recruited. Mean age (SD) were 56.2 (8.0) years for all participants, 55.5 (7.6) years for women, and 57.0 (8.3) years for men. Approximately 67% of the participants were illiterate and 76% were of Turkmen ethnicity. A total of 16.4% of the participants were current or past smokers, 17.6% reported past or current regular use of opium and 2.7% reported past or current regular alcohol use.

A total of 36 participants were excluded as their serum creatinine levels were either missing or outlying. A total of 11,373 observations were included in analyses.

Summary of baseline characteristics and laboratory data are presented in Table 1.

**Table 1:** Summary statistics of 11,409 GCS participants.

	Men (N = 5,413)	Women (N = 5,996)	All (N = 11,409)
	Mean (SD)	Mean (SD)	Mean (SD)
Age (year)	57.0 (8.3)	55.5 (7.6)	56.2 (8.0)
Weight (kg)	72.6 (14.4)	67.6 (14.3)	70.0 (14.6)
Height (m)	168.2 (6.6)	154.1 (5.7)	160.7 (9.4)
BMI (kg/m2)	25.6 (4.6)	28.5 (5.6)	27.1 (5.3)
Waist (cm)	93.3 (13.6)	95.7 (14.0)	94.6 (13.9)
Waist to Hip Ratio	0.95 (0.09)	0.96 (0.09)	0.95 (0.09)
Serum Creatinine	1.2 (0.3)	0.9 (0.2)	1.1 (0.3)
FBS (mg/dL)	103.1 (37.1)	106.3 (43.9)	104.8 (40.9)
HDL (mg/dL)	55.9 (13.3)	62.8 (15.2)	59.5 (14.7)
GFR (ml/min/1.73m^2^)	72.0 (15.0)	68.2 (14.2)	70.0 (14.7)

BMI: Body Mass Index; FBS: Fasting Blood Sugar; HDL: High Density Lipoprotein; GFR: Glomerular Filtration Rate

Mean (SD) of GFR was 70.0 ± 14.7 ml/min/1.73m^2^ among all participants, 68.2 ± 14.2 among women, and 72.0 ± 15.0 among men. Prevalence of CKD was 23.7% (26.6% in women, 20.6% in men). The percentage of CKD stages 3a, 3b, 4, and 5 were 20.0%, 3.3%, 0.35% and 0.10% respectively. Prevalence of hypertension and diabetes mellitus were 43.7% and 15.6% respectively. Prevalence of overweight and obesity were 37.1% and 27.0% respectively. About 13.3% of the participants had low HDL and 1,163 participants (10%) reported history of heart disease or stroke at repeated measurement. Prevalence of CKD and its 4 stages in demographic subgroups are presented in S1 Table. The differences in prevalence of CKD stages between men and women and the differences between all subgroups of determinants presented in S1 Table were statistically significant with p-values less than 0.001 (not shown). The p-value for trend was significant for BMI categories and there were no GFR stages 4 or 5 among underweight group.

### Determinants of CKD

We used logistic regression models with CKD as the outcome of interest. Results of the univariate and multivariate logistic regression models are demonstrated in Table 2. Sex had a significant association with CKD in both crude and adjusted models, with men being less likely to have CKD. Older age, urban residence, history of CVD, hypertension or diabetes, and low HDL were all associated with CKD. Being literate had inverse association. Ethnicity, smoking, and socio-economic status had no significant association, neither in crude and nor in adjusted models. Opium use exhibited inverse associations in crude model but as soon as sex was added to the model, the association became positive. Opium use was positively associated with CKD in the adjusted model. We found no significant association between history or incidence of any type of cancer with CKD (results not shown).

**Table 2:** The odds ratios for the association of determinants with CKD.

	Crude OR	Adjusted OR
Sex (male vs. female)	0.72 (0.66–0.78)	0.59 (0.53–0.66)
Age (year)	1.07 (1.06–1.08)	1.06 (1.05–1.07)
Residence (urban vs. rural)	2.91 (2.63–3.22)	2.37 (2.12–2.66)
Literacy (literate vs. illiterate)	1.01 (0.92–1.11)	0.76 (0.67–0.86)
CVD at baseline	2.35 (2.07–2.67)	1.47 (1.27–1.69)
Hypertension	2.26 (2.07–2.47)	1.58 (1.43–1.74)
Diabetes	1.57 (1.41–1.76)	1.09 (1.02–1.23)
HDL (low vs. high)	1.51 (1.34–1.70)	1.36 (1.20–1.55)
Opium ever use	0.87 (0.77–0.98)	1.24 (1.08–1.41)
BMI	-	-
Normal (18.5-<25 kg/m^2^)	1	1
Underweight (<18.5 kg/m^2^)	0.68 (0.54–0.85)	0.72 (0.57–0.91)
Overweight (25-<30 kg/m^2^)	1.41 (1.27–1.58)	1.30 (1.16–1.47)
Obese (> = 30 kg/m^2^)	1.60 (1.42–1.80)	1.37 (1.20–1.56)
Anthropometric measurements*		
BMI	1.04 (1.03–1.05)	
Waist circumference	1.02 (1.01–1.02)	-
Waist to Hip Ratio	1.40 (1.33–1.47)	-
Waist to Height Ratio	1.40 (1.33–1.46)	-

CKD was defined as eGFR < 60 ml/min/1.73m^2^.; BMI: Body Mass Index; CVD: Cardiovascular disease; HDL: High Density Lipoprotein;

*ORs are reported for every I unit increase in BMI or waist circumference and for every 0.1 units increase in WHR or WHT

**Fig 1:**
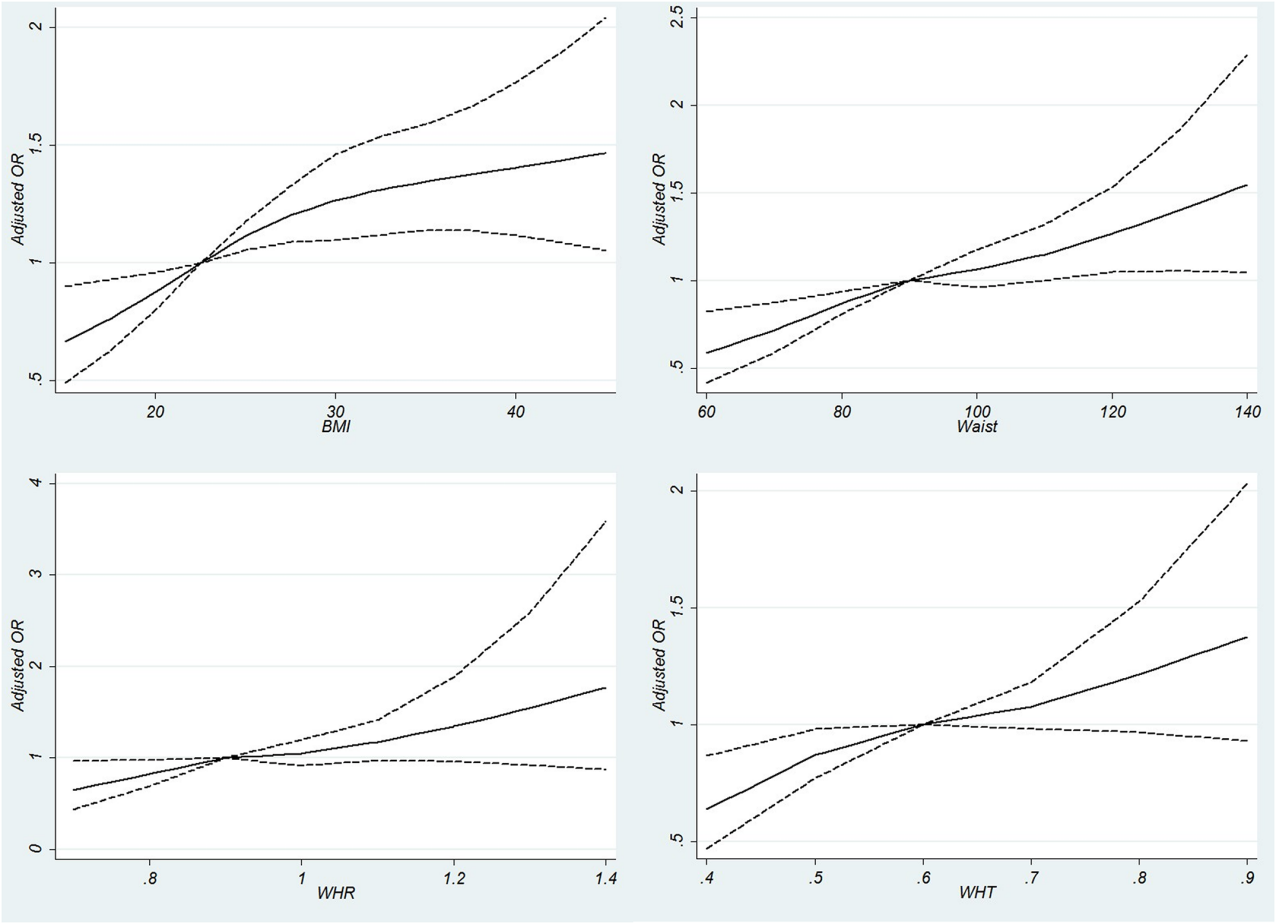
Association of anthropometric measurements with chronic kidney disease (CKD) defined as eGFR<60 ml/min/1.73m^2^ using restricted cubic splines. (A) Body Mass Index: using knots from 15 to 45 by 2.5 unit intervals and the reference point of 22.5 kg/m^2^. (B) Waist Circumference (cm) with knots ranging from 60 to 140 by 10 unit intervals and the reference point of 90 cm. (C) Waist to hip ratio (WHR): using knots ranging from 0.7 to 1.4 with 0.1 unit intervals and the reference point of 0.9. (D) Waist to height ratio (WHT): using knots ranging from 0.4 to 0.9 with 0.1 unit intervals and the reference point of 0.6.

**Fig 2:**
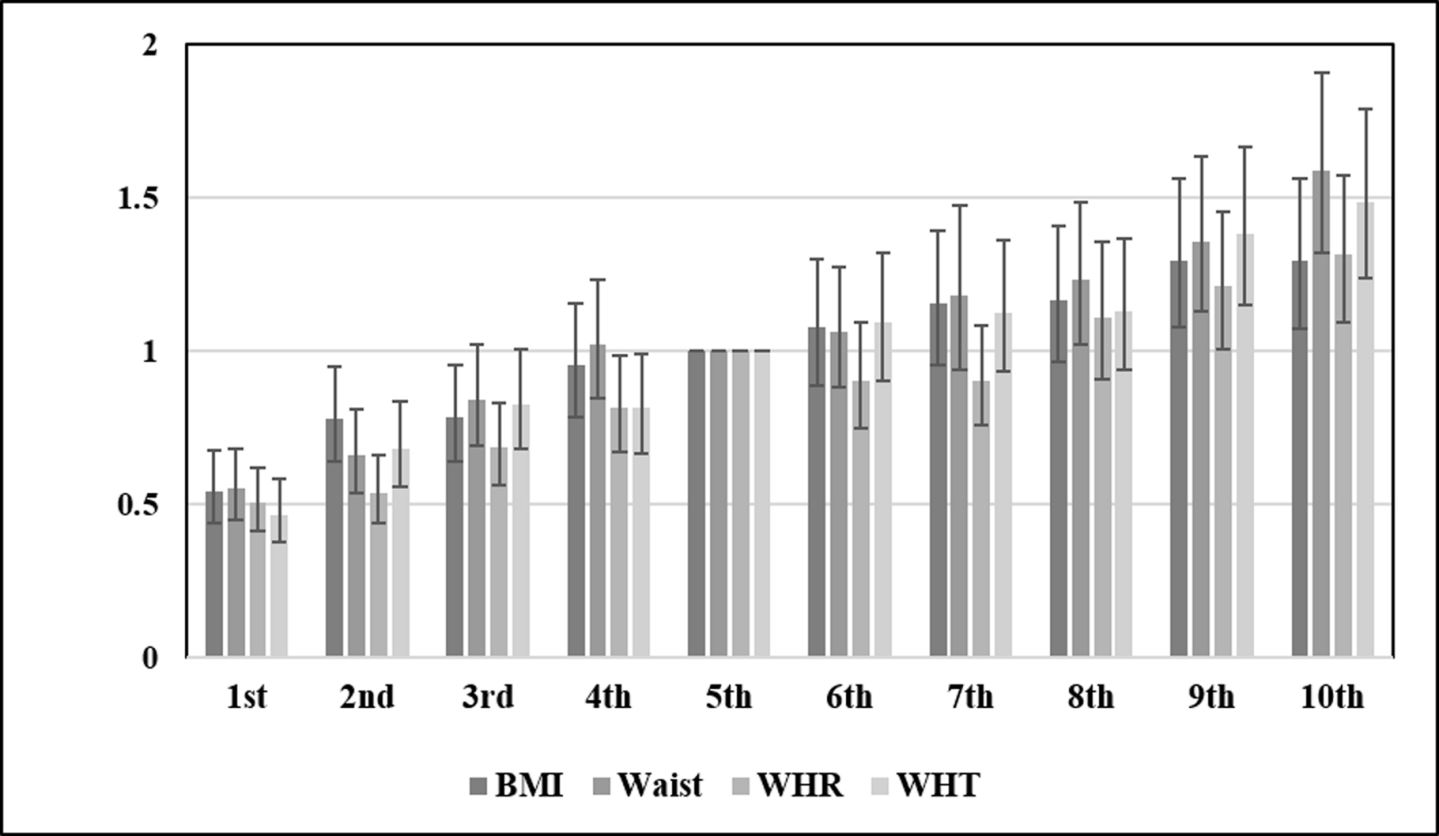
Comparing adjusted ORs for deciles of anthropometric measurements. The fifth decile serves as the reference group. Each decile includes approximately 1,100 participants. BMI: Body Mass Index, WHR: Waist to Hip Ratio, WHT: Waist to Height Ratio.

Anthropometric measurements including higher BMI, waist circumference, waist to hip ratio, and waist to height ratio were all associated with CKD. The adjusted ORs for categories of BMI are demonstrated in Table 2. The adjusted OR for waist circumference (for each 1 cm increase) was 1.01 (95% CI: 1.01–1.02). The adjusted ORs for each 0.1 unit increase in waist to hip ratio and waist to height ratio were 1.11 (95% CI: 1.03–1.20) and 1.13 (95% CI: 1.04–1.22) respectively. The ORs of covariates other than anthropometric indices were almost the same when BMI was replaced by alternative indices (not shown). Fig 1(A) to 1(D) demonstrates the adjusted ORs for the association of 4 anthropometric indicators with probability of CKD. As demonstrated in Fig 1, the association of anthropometric measurement with CKD is linear. It is important to note that sex had a significant interaction with all 4 anthropometric measurements. The interactions reported in Table 3 are built in the model. Therefore, the crude and adjusted ORs of anthropometric measurements were stratified by sex. As demonstrated in Table 3, anthropometric measurements have higher ORs among men compared to women. Fig 2 demonstrates the ORs for deciles of anthropometric measurements of BMI, waist circumference, WHR, and WHT. As demonstrated in the figure, the association of all anthropometric measurements with CKD is linear.

**Table 3:** Association of anthropometric measurements with chronic kidney disease stratified by sex.

	Crude OR	Adjusted OR*	P for Interaction
BMI (Kg/m^2^)			
Men	1.06 (1.04–1.08)	1.05 (1.04–1.07)	0.002
Women	1.02 (1.01–1.03)	1.03 (1.01–1.04)	
Waist (Cm)			
Men	1.03 (1.03–1.04)	1.03 (1.03–1.04)	0.021
Women	1.01 (1.01–1.02)	1.01 (1.01–1.02)	
WHR			
Men	1.46 (1.34–1.58)	1.25 (1.13–1.38)	0.008
Women	1.33 (1.25–1.42)	1.12 (1.03–1.22)	
WHT			
Men	1.49 (1.38–1.62)	1.28 (1.16–1.42)	0.014
Women	1.29 (1.21–1.37)	1.15 (1.06–1.24)	

BMI: Body Mass Index; WHR: Waist to Hip Ratio; WHT: Waist to Height Ratio; ORs are reported for every I unit increase in BMI or waist circumference and for every 0.1 units increase in WHR or WHT

*In multivariate model, ORs are adjusted for: age (continuous variable), residence, literacy, history of cardiovascular disease, hypertension, diabetes, low High Density Lipoprotein, and opium use. The interactions were built in the model.

**Fig 3:**
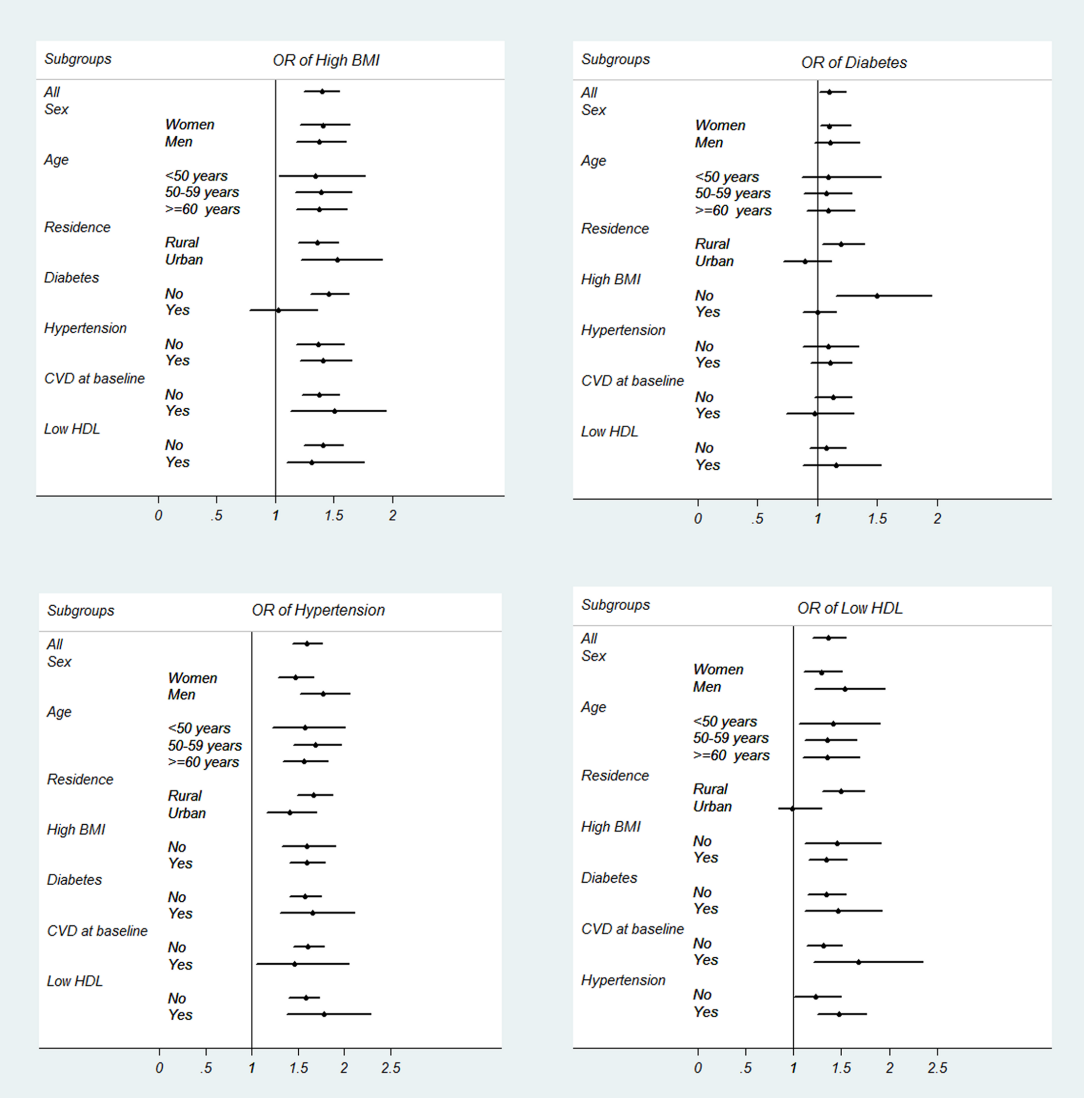
Odds ratios in subgroups. (A) Odds ratio of high BMI (> = 25 kg/m2) in association with CKD. (B) Odds ratio of diabetes (defined as fasting plasma glucose > = 126 mg/dL OR history of diabetes OR taking glucose lowering medications) in association with CKD. (C) Odds ratio of hypertension (defined as SBP/DBP > = 140/90 mmHg OR history of hypertension OR taking anti-hypertensive medications) in association with CKD. (D) Odds ratio of low HDL (defined as less than 40 mg/dL in men and less than 50 mg/dL in women) in association with CKD. *ORs are adjusted for sex, age, residence (urban vs. rural), literacy, diabetes, hypertension, history of cardiovascular disease, low HDL, and ever opium use, except for the covariate that is defined as the subgroup.

In Fig 3A to 3D, the association of high BMI, diabetes, hypertension, and low HDL with CKD is studied across subgroups. As demonstrated in the figure, the ORs for hypertension are all significantly positive across all subgroups. The OR of high BMI is also significantly positive across all subgroups, except for the subgroup of diabetics, among whom the OR becomes non-significant. The OR of low HDL is also significant across all subgroups except for urban dwellers. Diabetes however, has positive but non-significant ORs in most subgroups, except for the subgroup of low BMI among whom, the OR of diabetes in significantly positive.

**Fig 4:**
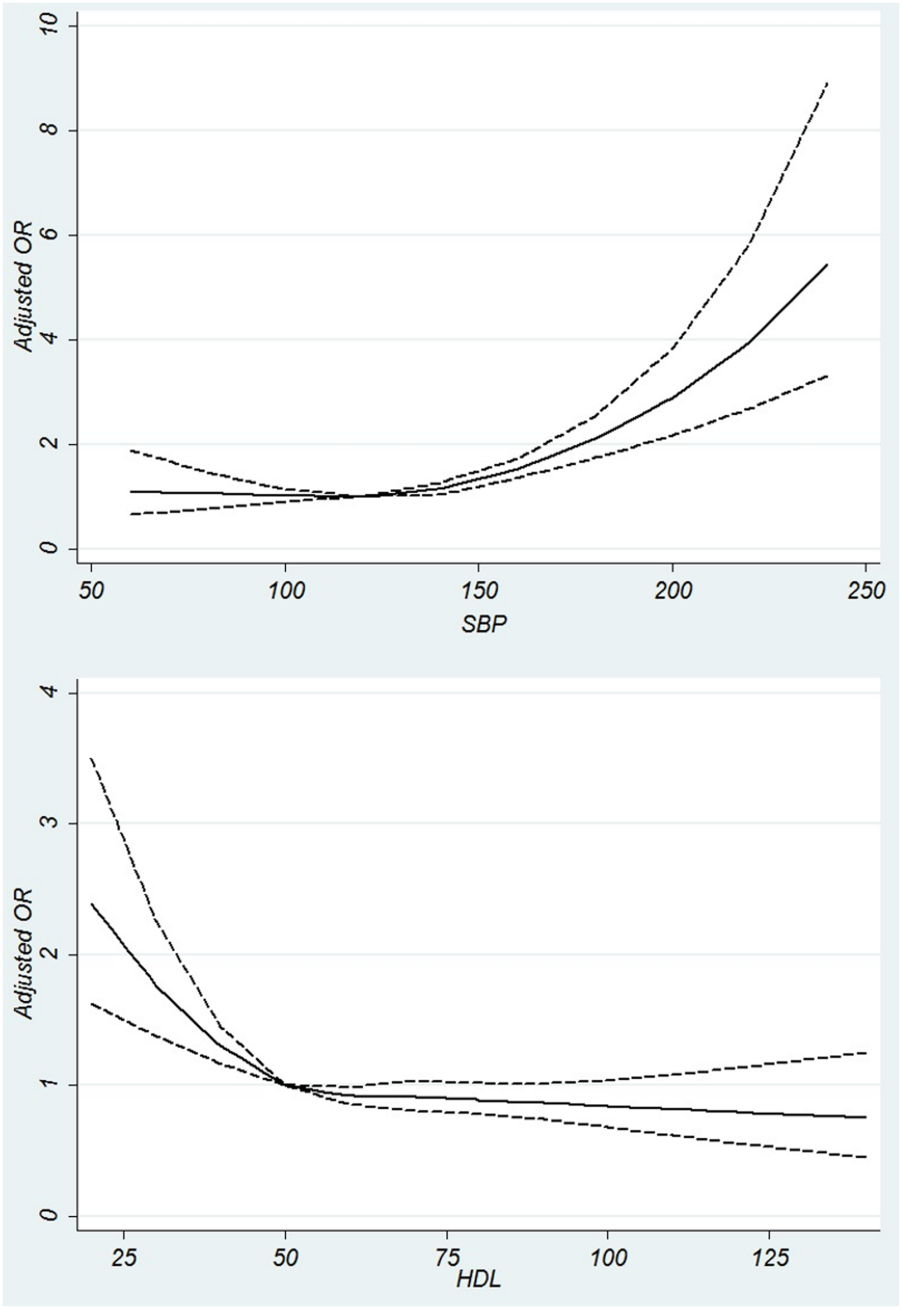
Association of systolic blood pressure and high density lipoprotein with chronic kidney disease. (A) Association of systolic blood pressure measurements with Chronic Kidney Disease using restricted cubic splines, with knots from 60 to 240 mmHg by 20 mmHg unit intervals and the reference point of 120 mmHg. (B) Association of High Density Lipoprotein (HDL) with Chronic Kidney Disease using restricted cubic splines, with knots from 20 to 140 mg/dL by 10 unit intervals and the reference point of 50 mg/dL.

Fig 4A and 4B demonstrate the adjusted ORs for the association of SBP and HDL as continuous covariates with probability of CKD. SBP shows a J-shaped association with CKD. HDL has a negative linear association with CKD.

### The association of rate of change in systolic blood pressure, BMI, and waist with CKD

In this model, instead of hypertension and waist circumference measured at repeated measurement, we used the level of systolic blood pressure (SBP), BMI, and waist circumference at baseline, as well as the rate of their change (change divided by the number of years of follow-up between baseline measurement and repeated measurement). Results show that the rate of change (each 10 mmHg increase per year for SBP, each one unit increase in BMI, and each one centimeter increase in waist circumference per year) had significant associations with CKD, independent of their initial level and with a higher effect size than their initial level (Table 4).

**Table 4:** Association of systolic blood pressure, body mass index, and waist circumference in main phase and the rate of their change from main phase to repeated measurement with chronic kidney disease defined as eGFR < 60 ml/min/1.73m^2^.

	Crude OR	Adjusted OR*	Adjusted OR*
SBP at baseline	1.19 (1.17–1.22)	1.07 (1.04–1.10)	1.07 (1.04–1.10)
Rate of change in SBP	1.65 (1.49–1.82)	1.20 (1.09–1.33)	1.20 (1.07–1.33)
BMI at baseline	1.05 (1.04–1.06)	1.02 (1.01–1.03)	-
Rate of change in BMI	1.06 (0.97–1.16)	1.20 (1.09–1.32)	-
Waist at baseline	1.01 (1.02–1.03)	-	1.01 (1.01–1.02)
Rate of change in waist	1.11 (1.08–1.15)	-	1.08 (1.04–1.11)

SBP: Systolic Blood Pressure; BMI: Body Mass Index; ORs are reported for each 10 mmHg increase in baseline SBP, each 10 mmHg increase in SBP per year, and each one unit increase in baseline BMI or waist circumference and each one unit increase per year.

*In multivariate models, ORs are adjusted for: sex, age, residence, literacy, history of self-reported cardiovascular disease and diabetes at baseline, opium use at baseline, taking anti-hypertensive medications and blood glucose lowering medications at baseline

## Discussion

In this large prospective cohort study from Middle East we demonstrated that one in four people in this cohort of general population with mean age (SD) of approximately 56 (8) years had low GFR. Female sex, older age, urban residence, history of CVD, hypertension or diabetes, and low LDL were all associated with CKD. Literacy showed an inverse association. All anthropometric measurements were positively associated with CKD and the association was stronger in men compared to women. Opium use was also a potent risk factor. The most outstanding result of the current study was to report a significantly high association between rate of change in waist circumference and systolic blood pressure with risk of CKD. This finding implies that interventional studies are warranted to examine whether halting the increase in waist and blood pressure may be as effective in reducing the excessive CKD burden.

Nafar et al conducted a systematic review and estimated that over 700,000 Iranian had CKD in 2004. [6] Safarinejad et al reported a prevalence of 12.6% for CKD among 17,000 adults over 14 years recruited from 2002 to 2005 in a cross-sectional study. [9] Najafi et al reported a prevalence of 4.6% for CKD based on GFR among adults > = 18 years in Golestan in 2008[7], which increased to 8.89% in their later report based on both GFR and albuminuria.[8] Naghibi et al reported a prevalence of 5.1% in Gonbad in 2012. [10] Our estimates imply a higher prevalence compared to previous reports. However, it is not possible to draw any significant conclusion regarding the trend of CKD prevalence in Iran as previous studies have been conducted in variable settings on participants in variable age ranges.

Our estimates are relatively higher than similar reports among adults in Western American and European countries. It should be noted however that our participants were above 43 years old at the time of this study and apparently, the mean eGFR in this group will be lower than the adult group over 25 years old. Additionally, the relative high prevalence of CKD in Golestan can be due to high prevalence of untreated hypertension and diabetes in this area as well as high opium use and high and rising prevalence of overweight and obesity. We were able to investigate the association of all of these risk factors with CKD in Golestan, which is one of the main merits of the current study.

The traditional risk factors for CKD including older age, hypertension, diabetes, history of cardiovascular disease, and low HDL were all confirmed in our study.[18–21] Unlike previous reports regarding J or U shape association of BMI and CKD, we observed a linear association. Interestingly, males had a lower odds of CKD than females. Literacy proved to be inversely associated with CKD while urbanization was associated with higher odds of CKD, and both associations remained statistically significant in multivariate analyses, which is consistent with previous reports.[7,8] Socio-economic status and ethnicity remained non-significant, neither in crude nor in adjusted models.

Our study had several novel findings. We found a strong association between opium use and CKD. Previous studies have reported a high rate of opium use among CKD patients but they had no control group.[22] Our results generate new hypotheses regarding the biologic plausibility of the association of opium intake with CKD, which requires more basic research. Previous studies in this same cohort have shown a higher risk of mortality from several causes, including cancer and cardiovascular causes, in association with opium use.[14]

The second highlighted result in our analyses was the stronger association of anthropometric measurements with CKD in men compared to women. This is in contrast with a number of previous reports on non-significance of interactions between sex and anthropometric measurements.[23]

The last novel result in our study is the association of rate of increase in SBP, BMI, or waist circumference with CKD, independent of their initial level. This finding may have important implications in both clinical practice and public health, as they suggest that preventing an increase in weight and blood pressure is as important as reducing current levels of weight and blood pressure.[24]

Our study has several potential limitations. First, this study has a cross-sectional design although it is embedded within the largest prospective study in Iran. Second, although the study sample is population based, it is evidently not representative of the entire nation and mostly captures the community of Turkmens in Iran. Third, we had to use traditional MDRD equation since the serum creatinine assay was not traceable to IDMS.[25] Fourth, we couldn’t do survival analyses as we hadn’t recorded the time of CKD onset and we couldn’t use the determinants for future projections. Fifth, we had measured serum creatinine only once and we couldn’t study its change from baseline to repeated measurement. And lastly, our estimates for CKD are not accurate enough due to unavailability of proteinuria.

## Conclusions

Considering the fact that Iran has a growing number of elderly and older age population and has an increasing trend of other CKD risk factors, such as obesity, hypertension, diabetes, and cardiovascular disease, this country is likely to face a high burden of CKD in near future. Primary prevention is essential. Policies should be made to increase the awareness of health care providers [26] as well as people towards early detection and prevention of CKD [27], which is essential for improving the prognosis of CKD, preventing the mortality and morbidity, and avoiding huge health care costs for end-stage renal disease. It is mandatory that screening programs and cost-effective guidelines be implemented at national level for primary and secondary prevention of CKD [20,28] and for proper definition of CKD.[29] Standardized equations and measurements can have significant added value for measurement and surveillance of asymptomatic low GFR.

## Supporting information

S1 Table The mean GFR, distribution of CKD in subgroups. (DOCX)
