# Supplementary material for: Prevalence and determinants of chronic kidney disease in northeast of Iran: Results of the Golestan cohort study
Source: PLoS One. 2017 May 3;12(5):e0176540. doi: 10.1371/journal.pone.0176540 (PMC5414986; doi:10.1371/journal.pone.0176540)
Supplement: S1 Table — (DOCX) [file pone.0176540.s001.docx]

**Appendix Table 1. The mean GFR, distribution of CKD in subgroups**

|  |  | GFR mean (SD) | CKD prevalence | Stages 3a and 3b | Stages 4 and 5 |
| --- | --- | --- | --- | --- | --- |
| Gender |  |  |  |  |  |
|  | Female | 68.2 (14.2) | 1,588 (26.6) | 1,553 (26.0) | 35 (0.59) |
|  | Male | 72.0 (15.0) | 1,112 (20.6) | 1,096 (20.3) | 16 (0.30) |
|  | Total | 70.0 (14.7) | 2,700 (23.7) | 2,649 (23.3) | 51 (0.45) |
| Age |  |  |  |  |  |
| 40-49 |  |  |  |  |  |
|  | Female | 72.9 (13.6) | 228 (14.2) | 224 (14.0) | 4 (0.25) |
|  | Male | 76.8 (14.5) | 129 (10.8) | 127 (10.7) | 2 (0.17) |
|  | Total | 74.6 (14.1) | 357 (12.8) | 351 (12.6) | 6 (0.21) |
| 50-59 |  |  |  |  |  |
|  | Female | 68.1 (13.5) | 664 (23.9) | 657 (23.6) | 7 (0.25) |
|  | Male | 72.7 (14.3) | 395 (16.4) | 389 (16.2) | 6 (0.25) |
|  | Total | 70.3 (14.1) | 1,059 (20.4) | 1,046 (20.2) | 13 (0.25) |
| 60+ |  |  |  |  |  |
|  | Female | 63.3 (14.4) | 696 (43.8) | 672 (42.2) | 24 (1.5) |
|  | Male | 67.8 (15.2) | 588 (32.8) | 580 (32.3) | 8 (0.45) |
|  | Total | 65.7 (15.0) | 1,284 (37.9) | 1,252 (37.0) | 32 (0.95) |
| Hypertension |  |  |  |  |  |
| No |  |  |  |  |  |
|  | Female | 70.5 (13.6) | 639 (20.2) | 634 (20.0) | 5 (0.16) |
|  | Male | 74.7 (14.3) | 468 (14.5) | 466 (14.4) | 2 (0.06) |
|  | Total | 72.6 (14.1) | 1,107 (17.3) | 1,100 (17.2) | 7 (0.11) |
| Yes |  |  |  |  |  |
|  | Female | 65.5 (14.4) | 949 (33.8) | 919 (32.7) | 30 (1.07) |
|  | Male | 67.9 (15.2) | 643 (29.8) | 629 (29.2) | 14 (0.65) |
|  | Total | 66.6 (14.8) | 1,592 (32.1) | 1,548 (31.2) | 44 (0.89) |
| Diabetes |  |  |  |  |  |
| No |  |  |  |  |  |
|  | Female | 68.7 (14.0) | 1,239 (25.1) | 1,218 (24.6) | 21 (0.42) |
|  | Male | 72.4 (14.9) | 909 (19.5) | 901 (19.4) | 8 (0.17) |
|  | Total | 70.5 (14.5) | 2,148 (22.4) | 2,119 (22.1) | 29 (0.30) |
| Yes |  |  |  |  |  |
|  | Female | 65.5 (15.0) | 349 (33.8) | 335 (32.5) | 14 (1.36) |
|  | Male | 69.0 (15.6) | 203 (27.5) | 195 (26.4) | 8 (1.08) |
|  | Total | 67.0 (15.3) | 552 (31.2) | 530 (29.9) | 22 (1.24) |
| HDL |  |  |  |  |  |
| High |  |  |  |  |  |
|  | Female | 68.5 (14.0) | 1,252 (25.5) | 1,229 (25.0) | 23 (0.47) |
|  | Male | 72.3 (14.9) | 983 (19.9) | 971 (19.6) | 12 (0.24) |
|  | Total | 70.4 (14.6) | 2,235 (22.7) | 2,200 (22.3) | 35 (0.36) |
| Low |  |  |  |  |  |
|  | Female | 66.5 (14.9) | 335 (31.5) | 324 (30.4) | 11 (1.03) |
|  | Male | 68.6 (15.7) | 128 (28.6) | 124 (27.7) | 4 (0.89) |
|  | Total | 67.1 (15.2) | 463 (30.6) | 448 (29.6) | 15 (0.99) |
| CVD |  |  |  |  |  |
| No |  |  |  |  |  |
|  | Female | 68.8 (14.0) | 1,338 (24.7) | 1,314 (24.3) | 24 (0.44) |
|  | Male | 72.7 (14.7) | 901 (18.8) | 892 (18.6) | 9 (0.19) |
|  | Total | 70.6 (14.5) | 2,239 (21.9) | 2,206 (21.6) | 33 (0.32) |
| Yes |  |  |  |  |  |
|  | Female | 62.0 (14.7) | 250 (44.1) | 239 (42.2) | 11 (1.94) |
|  | Male | 66.3 (16.3) | 211 (35.6) | 204 (34.5) | 7 (1.18) |
|  | Total | 64.2 (15.7) | 461 (39.9) | 443 (38.2) | 18 (1.55) |
| BMI |  |  |  |  |  |
| Underweight |  |  |  |  |  |
|  | Female | 72.3 (15.5) | 55 (19.5) | 55 (19.5) | 0 |
|  | Male | 78.4 (15.7) | 53 (11.1) | 53 (11.1) | 0 |
|  | Total | 76.1 (15.9) | 108 (14.2) | 108 (14.2) | 0 |
| Normal |  |  |  |  |  |
|  | Female | 69.5 (14.4) | 297 (22.2) | 289 (21.6) | 8 (0.60) |
|  | Male | 73.3 (14.9) | 355 (17.9) | 350 (17.6) | 5 (0.25) |
|  | Total | 71.8 (14.8) | 652 (19.6) | 639 (19.2) | 13 (0.39) |
| Overweight |  |  |  |  |  |
|  | Female | 67.8 (14.2) | 604 (28.0) | 591 (27.4) | 13 (0.60) |
|  | Male | 70.5 (14.5) | 476 (23.1) | 470 (22.8) | 6 (0.29) |
|  | Total | 69.1 (14.4) | 1,080 (25.6) | 1,061 (25.2) | 19 (0.45) |
| Obese |  |  |  |  |  |
|  | Female | 67.1 (13.8) | 632 (28.7) | 618 (28.1) | 14 (0.64) |
|  | Male | 68.9 (15.0) | 228 (26.4) | 223 (25.8) | 5 (0.58) |
|  | Total | 67.6 (14.2) | 860 (28.1) | 814 (27.4) | 19 (0.62) |

GFR: Glomerular Filtration Rate

CKD: Chronic Kidney Disease

HDL: High Density Lipoprotein

CVD: Cardiovascular Disease
